# Immunoglobulin Isotypes in Atlantic Salmon, *Salmo Salar*

**DOI:** 10.3390/biom5010166

**Published:** 2015-02-27

**Authors:** Ivar Hordvik

**Affiliations:** Department of Biology (BIO), High Technology Center, University of Bergen, Bergen N-5020, Norway; E-Mail: ivar.hordvik@bio.uib.no; Tel.: +47-5558-4538

**Keywords:** immunoglobulin, IgM, IgT, Atlantic salmon, tetraploidy, WGD

## Abstract

There are three major immunoglobulin (Ig) isotypes in *salmonid* fish: IgM, IgD and IgT, defined by the heavy chains μ, δ and τ, respectively. As a result of whole genome duplication in the ancestor of the *salmonid* fish family, Atlantic salmon (*Salmo salar*) possess two highly similar Ig heavy chain gene complexes (A and B), comprising two μ genes, two δ genes, three intact τ genes and five τ pseudogenes. The μA and μB genes correspond to two distinct sub-populations of serum IgM. The IgM-B sub-variant has a characteristic extra cysteine near the C-terminal part of the heavy chain and exhibits a higher degree of polymer disulfide cross-linking compared to IgM-A. The IgM-B:IgM-A ratio in serum is typically 60:40, but skewed ratios are also observed. The IgT isotype appears to be specialized to mucosal immune responses in *salmonid* fish. The concentration of IgT in serum is 100 to 1000 times lower than IgM. Secreted forms of IgD have been detected in rainbow trout, but not yet in Atlantic salmon.

## 1. Introduction

There are three major types of immunoglobulins in teleost fish: IgM, IgD and IgT/IgZ, the latter of which is unique for teleost fish [1]. The orthologous molecules in trout and zebrafish were published at the same time and named IgT and IgZ, respectively [2,3]; therefore the introduction of two different names. The monomer unit of each Ig type in teleost fish consists of two identical heavy chains and two identical light chains. Four types of Ig light chains have been characterized in teleost fish [1]. The isotype classification: IgM, IgD and IgT, is determined by the heavy chain of the molecule: μ, δ and τ, respectively.

**Figure 1:**
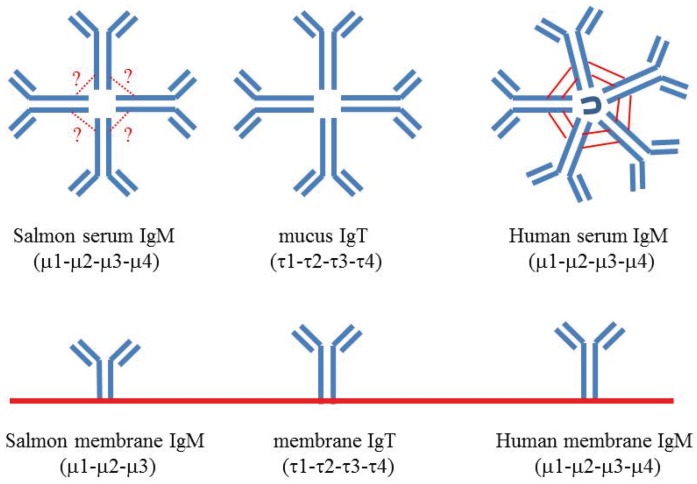
Schematic overview of secreted and membrane anchored IgM and IgT of salmonid fish, compared with human IgM. Serum IgM is a tetramer in teleosts. In contrast to the IgM pentamer in mammals, the monomer units in teleost IgM are loosely linked by disulfide bonding, and the J chain is lacking. Membrane anchored IgM in teleosts is one domain shorter than in mammals as a result of a special splicing pattern which excludes the entire fourth constant domain. Salmonid IgT appears to be non-covalently bonded tetramers in mucus. The similarity between the first constant domains of IgM and IgT is relatively high (52%) in Atlantic salmon, whereas the remaining domains of the heavy chains are only 13%–24% similar [7].

The IgM monomer has been relatively stable and maintained the primordial structure in all jawed vertebrates, but the primary amino acid sequence and the overall polymer structure has diverged considerably (Figure 1). The IgD molecule has been evolutionarily labile and has been subjected to many internal duplications and deletions [1]. Several mRNA splice variants of teleost δ have also been identified [4,5]. The ancestral δ gene arose very early in evolution and is orthologous to ω in elasmobranch fish [6]. The IgT/IgZ heavy chain in trout, salmon and zebrafish has four constant domains like IgM, but the number of τ domains varies among other teleosts [1].

The major systemic antibody in teleost fish is IgM, whereas IgT (at least in *salmonid* fish) is specialized to mucosal immune responses [7,8]. The IgT isotype in rainbow trout seems to be a monomer in blood and a tetramer in mucus [8]. Channel catfish, which apparently lack the IgT isotype, possess one δ gene, which encodes the membrane form, and another gene which encodes the secreted form [9]. Rainbow trout produce secreted and membrane anchored IgD by an alternative mRNA splicing strategy [5].

In mammals, the polymeric immunoglobulin receptor (pIgR) has a fundamental role in the transport of IgA (and IgM) across the epithelial cell layer into the mucus. A part of the pIgR (secretory component) is bound to the antibody and protects it from degradation in the hostile mucosal milieu. A molecule homologous to pIgR has been identified in teleosts and shown to be involved in transport of IgM [10]. However, teleost pIgR is quite different from the mammalian counterpart in structure: the secretory part is composed of two Ig domains, in contrast to five in mammals [11]. Using specific antibodies raised against the second Ig domain of rainbow trout pIgR (recombinant polypeptide) it was shown that IgT and IgM from gut mucus co-immunoprecipitate with the deduced secretory component of pIgR [8]. In salmon, a pIgR-like gene with an expression profile unlike pIgR has also been identified, and a cluster of pIgR-like genes was identified in zebrafish [11,12]. In zebrafish, pIgR-like molecules have shown to be involved in transport of phospholipids [12].

## 2. Immunoglobulin Heavy Chain Genes in Teleost Fish

The first identifications of immunoglobulin genes in teleost fish about 25 years ago were based on immunoscreening, DNA-cross-hybridization and PCR-homology cloning, and later, by large-scale sequencing and searches of sequence databases. Molecular characterization of IgM provided an explanation to a previous observation: that membrane bound IgM in teleost fish appeared to be shorter than the mammalian counterparts [13]. In contrast to mammals, where the first transmembrane exon (TM1) is spliced to a cryptic splice site in μ4, TM1 is spliced directly to μ3 in teleosts, excluding the entire μ4 exon [14]. Characterization of IgD transcripts from teleost fish revealed another special splicing pattern where μ1 is spliced to δ1 [15].

The Ig heavy chain genes in teleost fish have a translocon type of organization, and the δ gene is located immediately downstream of the μ gene like in higher vertebrates. However, the τ genes are located between variable gene segments upstream of μ and δ [16]. Accordingly, teleost fish do not have a class switch mechanism as in higher vertebrates (class switch means that a successfully recombined variable region gene can be expressed in the context of different classes of antibodies by a physical re-arrangement of constant region genes: for example by deletion of μ and δ and switch to γ1 during a secondary immune response; *i.e.*, an IgM to IgG1 switch).

The antibody repertoire in teleost fish is generated by V(D)J recombination of variable gene segments, enzymatic deletions or additions of nucleotides in the junctions, combinations of heavy and light chains, and subsequently, somatic mutations. Theoretically, there is a potential of creating an almost unlimited number of different antibodies, but it has been shown in humans that the usage of variable gene segments is not generally random. Some gene segments are used as little as 0.1%, whereas other are used more than 10%. As an outcome of evolution the expressed Ig repertoire is to some degree genetically determined [17], and it is reasonable to assume that there is a biased usage of variable gene fragments in teleost fish as well.

## 3. Ig Expression in Teleost Fish

Teleost fish have two primary lymphoid organs; the anterior part of the kidney (analogue of the bone marrow in mammals) and the thymus. The kidney serves as a secondary lymphoid organ as well, together with the spleen. The abundance of μ, τ and δ mRNA in teleosts is typically highest in head kidney and spleen, and correlates to a large degree with blood content in other tissues [7]. In a study of Atlantic salmon the quantity of μ transcripts was found to be more than 200 times that of δ, and τ transcripts were up to 20 times as abundant as δ [7]. At the protein level the IgM concentration in serum has been measured to be between 100 and 1000 fold higher than IgT in rainbow trout [8,18].

Fish possess a secondary capillary system (homologous to lymph vessels) but lack lymph nodes [19]. In mammals, the lymph nodes play an important role in the adaptive immune response; providing compartments for B and T cell communication during the adaptive immune response. In fish, clusters of melanomacrophages are believed to form the analogue of the germinal centers in mammalian lymph nodes. It is assumed that melanogenesis is an important immune mechanism in fish [20].

The gill is an extremely important entry point for pathogens. The gill constitutes more than 90% of the surface area of the fish and represents a semi-permeable barrier to the surrounding environment. In sea bass, the gill was identified as a major organ for antibody secreting cell production [21]. An intraepithelial lymphoid tissue (ILT) in the gills of Atlantic salmon was found to be rich in T cells whereas few IgM^+^ cells were found in the region that was examined [22,23,24]. However, it is likely that B cells are recruited to the gills during infection of *salmonid* fish, and that this is an arena for B and T cell communication. In mammals, mucosal surfaces harbor aggregations of organized lymphoid cells, *i.e.*, the tonsils in the pharynx and the Peyers patches of the intestines. The localization, architecture and arrangement of these immune tissues are of fundamental importance. In a study of rainbow trout it was found that the ratio of IgD to IgM in the gills was higher than in other tissues, indicating a significant role for IgD in the gills [5], and recently an IgD+IgM- B-lymphocyte subset was found to be mainly expressed in the gills [25]. The involvement of IgT in the gills needs further investigations. The IgT isotype has been found to be involved in mucosal immune responses in the intestine and on skin [8,26].

## 4. Ancestral Tetraploidy and Ig Sub-Variants in *Salmonid* Fish

Teleost fish is a very heterogenous group of animals; the number of species is estimated to be higher than 30,000. A whole genome duplication, which occurred early in the evolution of teleosts, more than 300 million years ago, has contributed to this diversity [27].

A second whole genome duplication event, estimated to approximately 96 million years ago, occurred in the ancestor of the *salmonid* fish family [28,29]. In spite of the long time period since this event, most of the duplicated DNA is maintained in present-day species of *salmonid* fish. Due to highly similar stretches of DNA in the haploid genome it has been a challenge to assemble the complete genome sequences of species like rainbow trout and Atlantic salmon. However, the genome sequence of rainbow trout was published recently [29], and the Atlantic salmon genome is underway.

There has been much speculation on how *salmonid* fish may benefit from their partially tetraploid genome, for example in their adaptation to an anadromous life cycle and various environments [28]. It is plausible to assume that some answers to major questions regarding timing and regulation can be found between the genes and in the overall structure of the DNA, rather than on protein coding loci. On the other hand, a high number of duplicated genes are still intact (approximately 50% in rainbow trout), several of which show differential mRNA expression patterns [29].

In agreement with ancestral tetraploidy there are two highly similar Ig heavy chain gene complexes, named A and B in the Atlantic salmon genome [30]. The A and B heavy chain gene complexes comprise two μ genes, two δ genes, three intact τ genes and five τ pseudogenes [16]. Expression of μA and μB is most likely regulated by a mechanism similar to allotypic exclusion.

The μA and μB genes correspond to two distinct IgM subpopulations in Atlantic salmon (IgM-A and IgM-B), which can be isolated from serum by gel filtration followed by anion exchange chromatography [31,32,33,34,35]. The IgM-B:IgM-A ratio in serum is typically 60:40, but skewed ratios are also observed [36]. An IgM-B:IgM-A ratio of 60:40 was found to be in accordance with the relative amounts of lymphocytes displaying IgM-B *versus* IgM-A on the surface [37].

**Figure 2:**
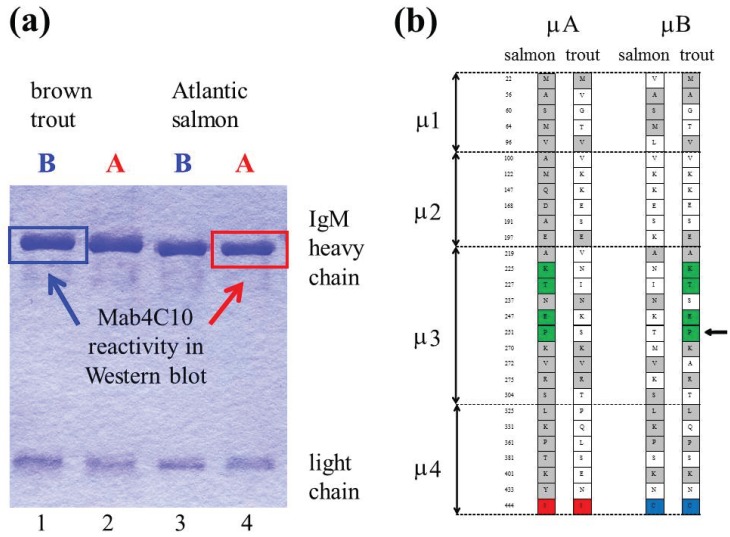
(**a**) A monoclonal antibody (Mab4C10) raised against rainbow trout IgM reacts exclusively with IgM-A in Atlantic salmon, whereas in brown trout it reacts with IgM-B. A coomassie stained gel of reduced IgM-B (lane 1) and IgM-A (lane 2) from brown trout, and IgM-B (lane 3) and IgM-A (lane 4) from salmon is shown (the lanes are indicated below the gel), and the reactivity with Mab4C10 is indicated with arrows (**b**) Schematic overview of amino acid substitutions in μ sub-variants of Atlantic salmon and brown trout. A cysteine residue near the C-terminal part of μ4 is characteristic for the μB sub-variant in Atlantic salmon and brown trout (indicated with blue). Residues which are identical in salmon μA and brown trout μB are indicated with green. By *in vitro* mutagenesis and transfection experiments it was shown that a proline (indicated with an arrow) in μ3 is essential for binding of Mab4C10 [35].

An extra cysteine residue near the C-terminal part of μB characterizes this sub-variant [33,34]. Comparative studies have shown that the IgM-B type is only present in species belonging to the *Salmo* genus, *i.e.*, Atlantic salmon and brown trout [35]. As illustrated in Figure 2a,b, a monoclonal antibody (Mab4C10) originally made against rainbow trout IgM have shown to react exclusively with IgM-A in Atlantic salmon, and with IgM-B in brown trout [34,35,38].

The fact that two Ig heavy chain gene complexes have been preserved in Atlantic salmon and brown trout during a long time in evolutionary terms, addresses questions regarding the biological significance of this phenomenon. Sequence analyses of the Atlantic salmon and brown trout μ genes indicate that recombination between the paralogous genes has occurred [34,35]. Recombination could have allowed one of the sub-variants to take over, but μA and μB have been conserved since the radiation of Atlantic salmon and brown trout. Divergence of both variable and constant region gene segments might be of significance regarding the maintenance of two parallel gene complexes in Atlantic salmon and brown trout. In a long-term perspective, the expected outcome of whole genome duplication is that one of the duplicated Ig heavy chain gene complexes is finally deleted after a process of recombination and fusion.

## 5. IgM Structure

Based on scanning electron microscopy photographs of IgM from some teleosts and estimation of the molecular weight of the polymers, IgM is regarded to be a tetramer in teleosts [31,39,40]. IgM is a pentamer in other groups of vertebrates and a J-chain is involved in the polymer formation of IgM (and IgA). A homolog of the J-chain appears to be absent in teleosts [41]. As illustrated by electron microscopy photographs, human IgM has a relatively flat ring structure, and when bound to a surface it can obtain a crab-like structure, changing the exposure of binding sites to other components of the immune system. Recently, a slightly modified IgM ring structure was predicted on the basis of a crystallographic structure of IgE Fc [42].

The 3D structure of teleost IgM is open to speculation. In contrast to mammals, where IgM cannot be secreted if it is not fully disulfide cross-linked, IgM tetramers of teleost fish are more loosely linked by disulfide bonding. In Atlantic salmon the degree of inter-heavy chain disulfide bonding appears to be higher in IgM-B than in IgM-A [35], presumably due to the extra cysteine residue near the C-terminal part of μB [33]. Accordingly, an additional cysteine residue in the C-terminal sequence of μ was found to be essential for establishing a series of covalently inter-bonded forms of IgM in channel catfish [43].

Our working hypothesis is that salmon IgM-A and IgM-B can be separated by anion exchange chromatography due to (1) distinct exposure of charged residues related to the polymer structure/post-translational modifications (e.g., glycosylation), and/or (2) a hitherto unidentified associated component. SDS-PAGE analyses have not revealed any additional bands that could indicate the presence of an associated molecule. However, an additional band might have been shaded by the Ig heavy or light chains. The Ig light chain bands vary extensively in size, and usually a spectrum of Ig light chains are seen by SDS-PAGE. Additional faint bands in the same region as the major Ig bands might represent posttranslational modifications.

In rainbow trout it has been shown that there is a correlation between greater antibody affinity and increased disulfide polymerization, *i.e.*, that high affinity B-cells produce more highly polymerized IgM, and that high-affinity highly polymerized antibodies possess longer half-lives than lower-affinity antibodies [44,45,46]. In this context, it is interesting that the IgM-B sub-variant in Atlantic salmon appears to be more highly polymerized than the IgM-A type.

## 6. Immunoglobulin Concentration in Serum

Although knowledge about Ig concentrations in fish is somewhat anecdotal there is no doubt that there are huge differences between species. The reported serum concentrations of IgM in fish vary to a great extent, depending on infection and vaccination status, and with several other parameters like, age/size/weight, gender, season, environment, *etc.* (Table 1).

**Table 1:** IgM concentration in serum of *salmonid* fish.

Species	Fish	Number of Fish	mg/mL Serum	Reference
Atlantic salmon	farm 2 to 8 kg	10	0.8 to 1.3	[31]
Atlantic salmon	farm 2.5 to 4 kg	2–3	~1	[40]
Brown trout	un-injected	104	7.4 ± 0.3	[47]
	PBS-injected	70	6.7 ± 0.2	[47]
	antigen-stimulated	399	7.5 to 16.8	[47]
Rainbow trout	stream	58	3.3 ± 2.1	[48]
	farm	25	2.2 ± 0.7	[48]
	farm VHS/ERM	40	4.2 ± 1.5	[48]
	farm PKD	10	10.9 ± 5.3	[48]
	aquarium	20	1.5 ± 0.4	[48]
Rainbow trout	20 g (temp 19 °C)	10	0.67 ± 0.66	[49]
	125 g (temp 19 °C)	15	9.1 ± 4.17	[49]
	>1 kg (temp 19 °C)	12	9.36 ± 2.78	[49]
	>1 kg (temp 7 °C)	12	3.51 ± 1.18	[49]
Masu salmon	larvae 88–235 dph	100 + 50 (pooled)	<0.1	[50]
	251–429 dph	59	0.69 ± 0.037	[50]
	adult males	108	2.273 ± 118	[50]
	adult females	125	2.049 ± 97	[50]
Coho salmon	50–200 g	3 groups	2.1 ± 0.1	[51]

VHS = viral haemorrhagic septicaemia; ERM = enteric red-mouth; PKD = proliferative kidney disease; dph = days post hatch.

**Figure 3:**
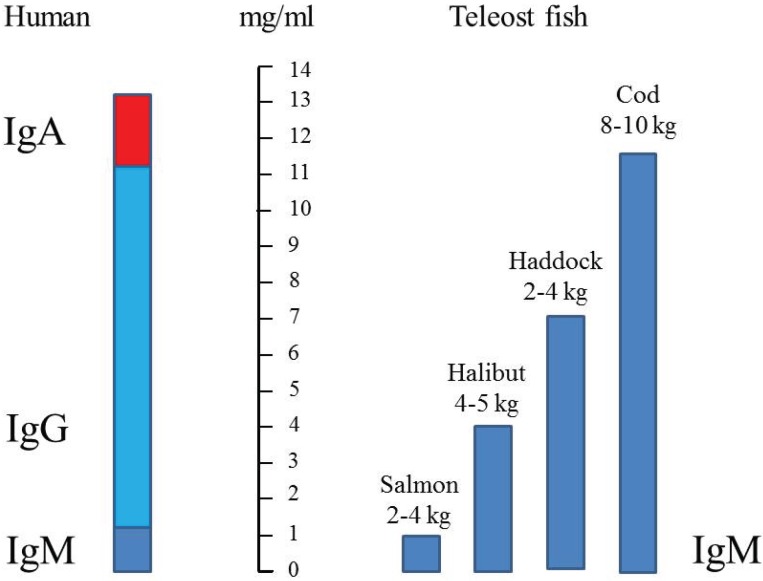
Comparison of serum immunoglobulin (IgM) concentrations in different teleosts [40] compared to human (IgM + IgG + IgA). IgM is indicated with dark blue, IgG with light blue and IgA with red.

In some recent reports it has been shown that Atlantic salmon that have received commercial oil-adjuvanted vaccines have IgM concentrations that are two- to fourfold higher than in un-vaccinated fish. The depot effect of the mineral oil seems to trigger a continuous higher production of IgM which persists to the fish are slaughtered [36,52]. As shown in Table 1, a study of rainbow trout revealed an average serum IgM concentration of 3.3 mg/mL in free living fish [48]. When the fish were transferred to an aquarium the concentration dropped to 1.5 mg/mL. Fish from farms with outbreaks of VHS and ERM disease had average IgM concentrations of 4.2 mg/mL, and in a farm with PKD it was 10.9 mg/mL [48]. In a recent study of rainbow trout, the IgM concentration was found to increase 15 fold during systemic VHSV infection [18].

Magnadottir [40] did a comparison of four different species, and found that serum IgM concentration in Atlantic salmon (~1 mg/mL) was significantly lower than in other species (Figure 3). This estimate is in agreement with measurements done in our laboratory [31].

## 7. Conclusions

Exciting discoveries of IgD and IgT responses in mucosal tissues of *salmonid* fish have attracted much focus these last years [5,8,25,26]. IgT can be highly up-regulated in response to pathogens [8,18]. However, the amount of IgT produced is not comparable to IgA in mammals, and the concentration in serum is very low (estimated to 3.7 μg/mL in a study of rainbow trout [8]). Elucidating the impact of mucosal antibody responses (and the mechanisms of communication between mucosal and systemic components) is of great interest regarding alternative delivery regimes of vaccines to fish, as well as in basic immunology research. As indicated by this review many basic questions regarding the major serum immunoglobulins are still not resolved. Several studies of immunoglobulins were performed before the discoveries of IgD and IgT, and in the belief that IgM was the only isotype present in teleost fish. A retrospective survey of early literature might be useful and inspire new studies with the improved toolbox available today.
